# Coronary Flow, Left Ventricular Contractile and Heart Rate Reserve in Non-Ischemic Heart Failure

**DOI:** 10.3390/jcm10153405

**Published:** 2021-07-30

**Authors:** Clarissa Borguezan Daros, Quirino Ciampi, Lauro Cortigiani, Nicola Gaibazzi, Fausto Rigo, Karina Wierzbowska-Drabik, Jaroslaw D. Kasprzak, Claudio Dodi, Bruno Villari, Francesco Antonini-Canterin, Valentina Lorenzoni, Michele De Nes, Clara Carpeggiani, Eugenio Picano, on behalf of the Stress Echo Study Group of the Italian Society of Echocardiography and Cardiovascular Imaging

**Affiliations:** 1 Cardiology Division, Hospital San José, Criciuma 88801-250, Brazil; clarissabdaros@cardiol.br; 2 Cardiology Division, Fatebenefratelli Hospital, 82100 Benevento, Italy; villari.bruno@gmail.com; 3 Cardiology Department, San Luca Hospital, 55100 Lucca, Italy; lacortig@tin.it; 4 Cardiology Department, Parma University Hospital, 43100 Parma, Italy; ngaibazzi@gmail.com; 5 Department of Cardiology, Dolo Hospital, 30031 Venice, Italy; faustorigo@alice.it; 6 Chair of Cardiology, Bieganski Hospital, Medical University, 90-647 Lodz, Poland; wierzbowska@ptkardio.pl (K.W.-D.); kasprzak@ptkardio.pl (J.D.K.); 7 Casa di Cura Figlie di San Camillo, 26100 Cremona, Italy; claudio.dodi@gmail.com; 8 Cardiac Prevention and Rehabilitation Unit, Highly Specialized Rehabilitation Hospital Motta di Livenza, 31045 Motta di Livenza, Italy; antonini.canterin@gmail.com; 9 Institute of Management, Scuola Superiore Sant’Anna, 56100 Pisa, Italy; v.lorenzoni@sssup.it; 10 CNR, Biomedicine Department, Institute of Clinical Physiology, 56100 Pisa, Italy; denesm@ifc.cnr.it (M.D.N.); claracarpeggiani@gmail.com (C.C.); picano@ifc.cnr.it (E.P.)

**Keywords:** chronotropic incompetence, coronary flow velocity reserve, heart failure, stress echocardiography

## Abstract

**Background**: Left ventricular contractile reserve (LVCR), coronary flow velocity reserve (CFVR), and heart rate reserve (HRR) affect outcome in heart failure (HF). They can be simultaneously measured during dipyridamole stress echocardiography (DSE). **Aim**: To assess the value of comprehensive DSE in patients with non-ischemic HF. **Methods**: We evaluated 610 patients with HF, no history of coronary artery disease, and no inducible regional wall motion abnormalities: 270 patients with preserved ejection fraction (≥50%), 146 patients with mid-range ejection fraction (40–49%), and 194 patients with reduced ejection fraction (<40%). All underwent DSE (0.84 mg/kg in 6′) in 7 accredited laboratories. We measured LVCR (abnormal value ≤ 1.1), CFVR in left anterior descending artery (abnormal value: ≤2.0), and HRR (peak/rest heart rate; abnormal value: ≤1.22). All patients were followed up. **Results**: Abnormal CFVR, LVCR, and HRR occurred in 29%, 45%, and 47% of patients, respectively (*p* < 0.001). After a median follow-up time of 20 months (interquartile range: 12–32 months), 113 hard events occurred in 105 patients with 41 deaths, 8 myocardial infarctions, 61 admissions for acute HF, and 3 strokes. The annual mortality rates were 0.8% in 200 patients with none abnormal criteria, 1.8% in 184 patients with 1 abnormal criterion, 7.1% in 130 patients with 2 abnormal criteria, 7.5% in 96 patients with 3 abnormal criteria. **Conclusions**: Abnormal LVCR, CFVR, and HRR were frequent during DSE in non-ischemic HF patients. They target different pathophysiological vulnerabilities (myocardial function, coronary microcirculation, and cardiac autonomic balance) and are useful for outcome prediction.

## 1. Introduction

Heart failure (HF) affects 10% of patients aged 70 years and over. Due to the heterogeneity of the disease, phenotyping and risk stratification remain a challenge [1]. For all values of ejection fraction (EF), the functional characterization may show abnormalities of myocardial function, coronary microcirculation, and the cardiac autonomic system. These abnormalities can also appear in the absence of underlying coronary artery disease (CAD) and are possibly involved in HF initiation and/or progression [1]. Dipyridamole stress echocardiography (DSE) measures left ventricular contractile reserve (LVCR) for the assessment of myocardial function [2], coronary flow velocity reserve (CFVR) for coronary microcirculation [3], and heart rate reserve (HRR) for the cardiac sympathetic nervous system [4]. Each functional abnormality may contribute to the phenotypic and prognostic heterogeneity of HF and is a specific potential therapeutic target, represented by different cell types, such as cardiomyocytes for LVCR [5], coronary small vessels for CFVR [6], and neurons for HRR [7].

The hypothesis in the present study was that LVCR, CFVR, and HRR may have complementary prognostic value in patients with non-ischemic HF for all values of resting EF. To test this hypothesis, we interrogated the data bank of multicenter stress echocardiography studies built over the last 20 years [8].

## 2. Materials and Methods

### 2.1. Study Population

From 2004 to 2019, data from patients with known or suspected HF referred to DSE were prospectively collected and retrospectively analyzed. The population eventually enrolled consisted of 610 patients with non-ischemic HF recruited by 7 centers (Benevento, Cremona, Criciuma, Lucca, Parma, Venezia, and Lodz) from 3 countries (Brazil, Italy, and Poland). All patients underwent DSE testing as part of a clinically driven work-up according to the referring physician’s indications and entered a regular follow-up program as part of the clinical routine in these centers. Inclusion criteria were as following: (1) age: >18 years; (2) known or suspected HF, with any degree of resting global left ventricular (LV) function (preserved or reduced), defined according to the criteria of the European Society of Cardiology [1]; (3) no severe primary valvular or congenital heart disease or hypertrophic cardiomyopathy; (4) transthoracic echocardiography (TTE) of sufficient quality at rest (<2 uninterpretable segments of left ventricle); (5) written informed consent.

Exclusion criteria were as following: (1) history of CAD: previous myocardial infarction, and/or previous myocardial revascularization (with coronary bypass or percutaneous coronary intervention) and/or significant CAD at noninvasive or invasive coronary angiography; (2) unfeasible and/or uninterpretable Doppler tracings for CFVR assessment; (3) stress-induced regional wall motion abnormalities (RWMA) during DSE (indicating true inducible ischemia possibly for angiographically occult CAD missed by coronary angiography); (4) patients recruited by centers without structured follow-up programs.

The study protocol was reviewed and approved by the institutional ethics committees as part of the Stress echo 2020 study (148-Comitato Etico Lazio-1, on 16 July 2016; Clinical trials. Gov Identifier NCT 030.49995).

### 2.2. Stress Echocardiography

Resting TTE and DSE (0.84 mg/kg over 6 min) were performed according to standard recommendations [9,10] as previously detailed [8]. One reader from each lab passed the training and certification process through a web-based system prior to starting recruitment. The wall motion score index (WMSI) was calculated with a four-point score ranging from 1 (normal) to 4 (dyskinetic) in a 17-segment model of the left ventricle [11]. LVCR (step C) was calculated as the stress/rest ratio of force (systolic blood pressure/end-systolic volume), and a value of ≤1.1 was considered abnormal (positive) [2]. Systolic blood pressure was obtained by a cuff sphygmomanometer, an end-systolic volume from apical biplane views of the left ventricle, and Simpson’s rule. CFVR (step D) was assessed in the mid-distal portion of the left anterior descending coronary artery by pulsed-wave Doppler of the peak diastolic flow velocity, and a value of ≤2.0 was considered abnormal (positive) [3,4,5,6,7,8,9,10,11,12]. All studies were digitally stored to simplify offline reviews and measurements. At each time point, three optimal profiles of peak diastolic Doppler flow velocities were measured, and the results were averaged.

HRR (step E) was calculated as the peak/rest hazard ratio (HR) from 12-lead electrocardiogram (EKG) [4]. It was abnormal in the presence of HRRs of ≤1.22 [4] or ≤1.17 with permanent atrial fibrillation [13]. HRR was retrievable, since the information on rest and peak HR was part of the standard protocol and the minimum data set required for entering the studies in the data bank. The excellent inter-observer and intra-observer reproducibility of CFVR (>95%) among experienced observers has been previously documented [8]. Feasibility and reproducibility are 100% for HRR [8].

DSE response was summarized with a score ranging from 0 to 3 as follows: score 0 (all markers within normal limits) or scores 1–3, according to the number of abnormal steps (e.g., score 3 indicated all 3 steps of LVCR, CFVR, and HRR were abnormal).

### 2.3. Outcome Data Analysis

Follow-up data were obtained from the national health service database by assessors unaware of test results or by review of the patient’s hospital record, personal communication with the patient’s physician and review of the patient’s chart, a telephone interview with the patient or a patient’s close relative conducted by trained personnel, or a staff physician visiting the patients at regular intervals in the out-patient clinic. Mortality was the primary end-point. In order to avoid the misclassification of the cause of death, overall mortality was considered.

We also considered a composite secondary end-point of all-cause death, new hospital admissions for acute HF, non-fatal myocardial infarction, and non-fatal stroke (one composite end-point per patient).

### 2.4. Statistical Analysis

Categorical data are expressed in terms of number of subjects and percentage, while continuous data are expressed as mean ± standard deviation or median (minimum–maximum), depending on variables’ distribution. For continuous variables, intergroup differences were tested with one-way analysis of variance. Chi-square tests or Fisher exact tests were used to compare the distribution of categorical variables among the groups. Survival was estimated using the Kaplan–Meier method, and survival curves were compared by means of the Logrank test. The univariate Cox proportional hazards model were used to identify candidate predictors for selected endpoints. All variables with *p* of <0.10 at univariate analysis were considered for the inclusion in the multivariate Cox proportional hazards model. Collinearity was verified for all the models. We used the variance inflation factors to check the presence of collinearity and values for all the variables were below 1.4, thus suggesting no evidence of collinearity in the model.

The incremental value of each DSE variable (LVCR, CFVR, and HRR) was evaluated by comparing multivariable models with and without individual steps using global X2 values to evaluate the improvement of goodness-of-fit. Statistical significance was set at *p* < 0.05. All analyses were performed using STATA (STATACorp. Stata statistical software was Release 14. College Station, TX: STATACorp LP and R version 3.6.)

## 3. Results

The patients’ characteristics for the overall population are shown in Table 1 and also split into three subsets on the basis of resting EF values: group 1 with preserved EF (HFpEF; ≥50%); group 2 with mid-range EF (HFmrEF; from 40% to 49%); group 3 with reduced EF (HFrEF; <40%). The patients in group 1 were older, more frequently males and hypertensives, compared to those in groups 2 and 3 (Table 1).

### 3.1. DSE Findings

**Figure 1:**
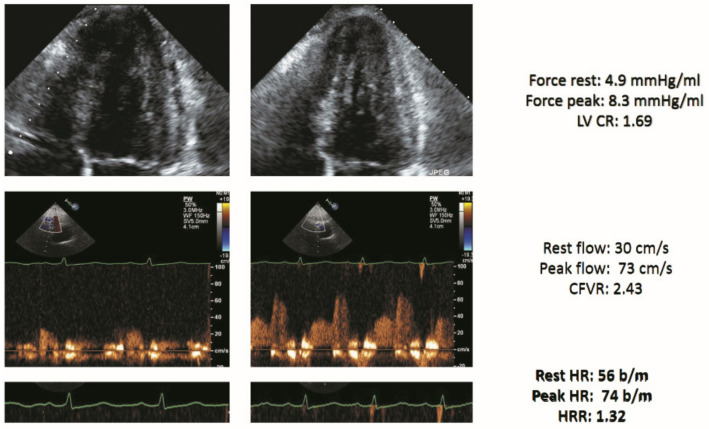
Stress echo with a normal score of 0 in a patient with heart failure (HF). Left column: rest. Right column, stress. From top to bottom: normal end-systolic volume at rest which decreased during stress (force rest = 4.9 mm Hg/mL; stress = 8.3 mm Hg/mL) with a normal LVCR (1.69); normal increase of pulsed-wave Doppler peak diastolic flow (rest = 30 cm/s; stress = 73 cm/s; CFVR = 2.43); normal heart rate reserve: heart rate rest = 56; peak = 74 bpm; heart rate reserve (74/56) = 1.32. Abbreviations: CFVR, coronary flow velocity reserve; EKG, electrocardiogram; HRR, heart rate reserve; LAD, left anterior descending coronary artery; LV, left ventricle; LVCR, left ventricular contractile reserve.

An example of a negative DSE result with triple biomarker assessment is shown in Figure 1.

**Figure 2:**
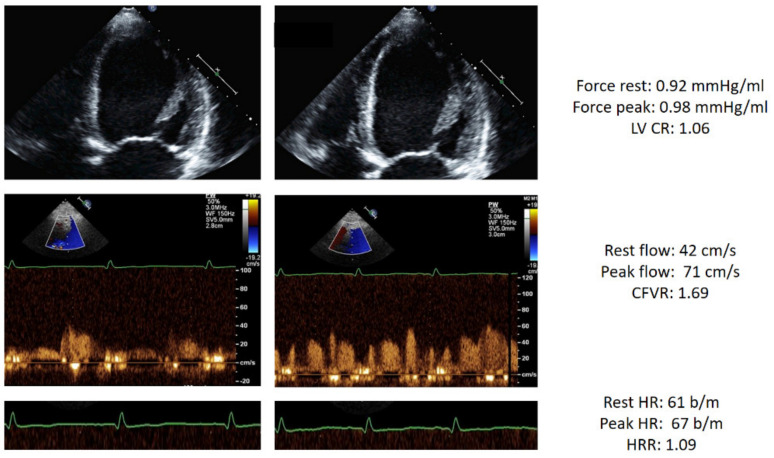
Stress echo with abnormal score 3 in a patient with HF. Left column: rest. Right column, stress. From top to bottom: dilated end-systolic volume at rest which does not decrease during stress (force rest = 0.92 mm Hg/mL; stress = 0.96 mm Hg/mL) with abnormal LVCR (1.06); blunted increase of pulsed-wave Doppler peak diastolic flow (rest = 42 cm/s; stress = 71 cm/s; CFVR = 1.69); abnormal heart rate reserve: heart rate rest = 61; peak = 67 bpm; heart rate reserve (67/61) = 1.09. Abbreviations: CFVR, coronary flow velocity reserve; EKG, electrocardiogram; HRR, heart rate reserve; LAD, left anterior descending coronary artery; LV, left ventricle; LVCR, left ventricular contractile reserve.

An example of a triple positivity of DSE result is shown in Figure 2.

The main DSE findings in the three groups are reported in Table 2.

The predictors of the abnormal C step (reduced LVCR) was as following: reduced EF (Odds ratio (OR): 1.757; 95% confidence interval (CI): 1.335–2.312; *p* < 0.001). The predictors of the abnormal D step (reduced CFVR) were as following: reduced EF (OR: 1.976; 95% CI: 1.387–2.816; *p* < 0.001) and hypertension (OR: 1.586; 95% CI: 1.163–2.164; *p* = 0.004). The predictors of the abnormal E step (reduced HRR) were as following: reduced EF (OR: 3.274; 95% CI: 2.368–4.528; *p* < 0.001) and hypertension (OR: 1.465; 95% CI: 1.149–1.869; *p* = 0.002).

**Figure 3:**
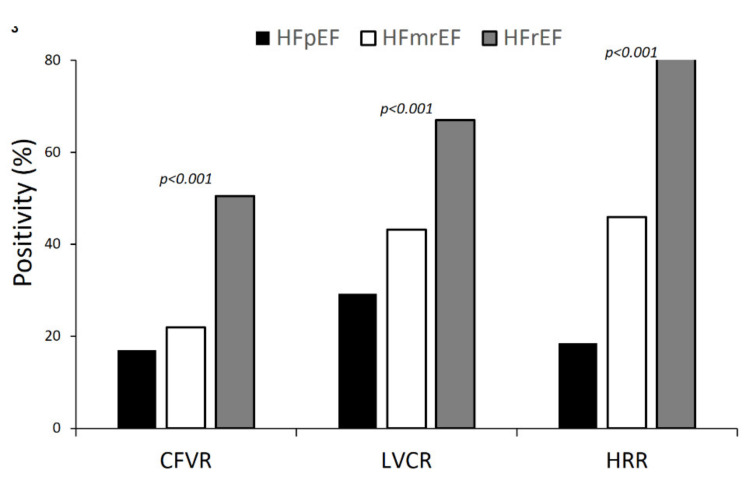
Dipyridamole stress echocardiography (DSE) results: positivity rate for each of the 3 parameters (LVCR, CFVR, and HRR) in the 3 groups (i.e., HFpEF, HFmrEF, and HFrEF). All intergroup differences are significant.

Abnormalities of LVCR, CFVR, and HRR abnormalities were more prevalent in patients with HFrEF when compared to in those with HFmrEF and HFpEF (*p* < 0.001) (Figure 3).

### 3.2. Outcome Data Results

After a median follow-up time of 20 months (interquartile range: 12–32 months), 113 hard events occurred in 105 patients: 41 deaths, 8 myocardial infarctions, 61 admissions for acute HF, and 3 strokes.

For the primary end-point of all-cause death, a reduced CFVR (HR = 4.071; 95% CI: 1.643–10.087; *p* = 0.002) was the only independent predictor (Table 3).

**Figure 4:**
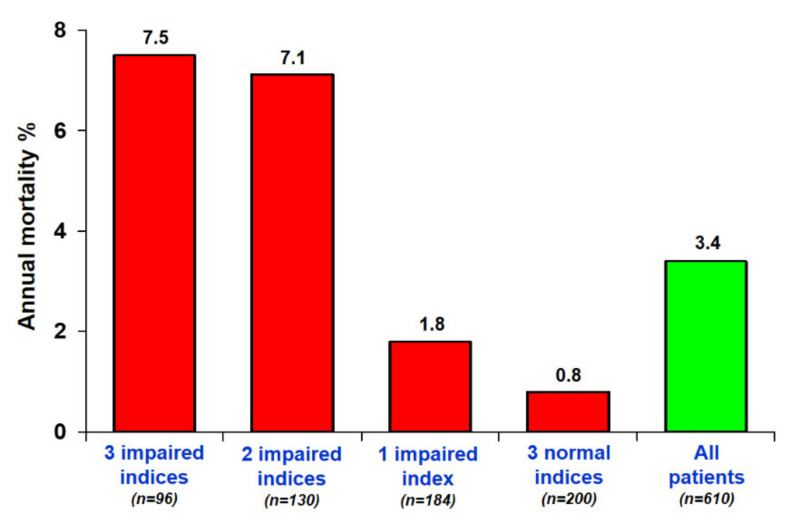
Annual mortality rates and numbers of positivity criteria. The annual mortality rate is highest in patients with the highest SE score.

Five-year mortality was 10 times higher (7.5% vs. 0.8%; *p* < 0.0001) in patients with score 3 compared to those with score 0 (Figure 4).

For composite end-points, the three reserves (LVCR, CFVR, and HRR) each showed an independent value for predicting events at multivariable analysis (Table 4).

**Figure 5:**
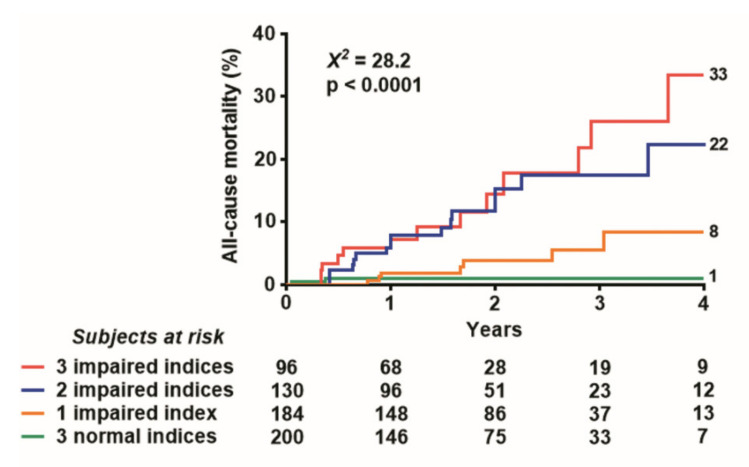
Event-free Kaplan–Meier survival curves. The five-year event-free survival is lowest in patients with the highest SE score.

Event-free survival worsened with increasing values of the DSE score (Figure 5).

At incremental analysis, the global X2 of the clinical model for the prediction of death increased considering clinical variables positive at univariate analysis (age, male sex, diabetes, and resting EF) to progressively higher values with the addition of DSE variables of LVCR (global chi-square = 12.6; *p* = 0.005), followed by CFVR (global chi-square = 19.5; *p* = 0.001) and by HRR (global chi-square = 28.2; *p* < 0.001), when each variable was added to the previous one.

When we considered all cause of death combined with acute HF, the best predictors were as following: abnormal step D (reduced CFVR): (HR: 3.592; 95% CI: 2.285–5.647; *p* < 0.001) and abnormal E (reduced HRR): (HR: 2.019; 95% CI: 1.176–3.467; *p* = 0.011).

## 4. Discussion

Patients with HF showed abnormalities of coronary microvascular, left ven-tricular contractile, and cardiac autonomic reserve during DSE, mirrored by abnormal values of CFVR (in 29% of patients), LVCR (45%), and HRR (47%). These abnormalities were detectable across all values of resting EF but are 2-to-3-fold more prevalent in HFrEF patients compared to in HFpEF patients. Each abnormality provided an independent and incremental value in predicting outcome, and the annual mortality was 0.8% in patients with all parameters normal up to 7.5% (9-fold higher) in patients with all three parameters abnormal.

Patients with HF have multiple functional abnormalities and potential prognostic vulnerabilities, not mutually exclusive and largely independent from one another. DSE allows detecting abnormal LVCR, CFVR, and HRR, which identify distinct phenotypes, contribute to better risk stratification and may represent separate therapeutic targets. They are well known pathophysiological hallmarks of the complex syndrome of HF. The morphological and functional correlation of a reduced LVCR is the increased myocardial fibrosis or necrosis of the left ventricle [14]. The main mechanism underlying coronary microvascular alterations is the increased oxidative stress and low-grade inflammation toxic for endothelium and smooth muscle cells of coronary micro-vessels [15]. Chronotropic incompetence can be due to reduced sensitivity of beta-1 adrenergic receptors present on the sinus node to increased noradrenaline of the neuronal origin evoked by endogenous adenosine accumulation during dipyridamole infusion [16]. Each mechanism can be theoretically targeted by focused interventions such as cardiac contractility modulation for myocardial abnormalities, angiotensin-converting enzyme inhibitors or statins for coronary microvascular abnormalities [17], and rate-responsive pacemakers for chronotropic incompetence [18]. The recognition of personalized phenotypes is important for a tailored therapy, since the unrecognized functional heterogeneity of HF may lead to unsatisfactory response to therapy.

### 4.1. Comparison with Previous Studies

The observed prevalence of abnormal LVCR, CFVR, and HRR was higher in presence of lower resting values of EF, as shown by previous studies using different imaging and stress modalities [19]. The independent and incremental values of the C (LVCR), D (CFVR) and E (HRR) steps of the state-of-art DSE protocol have also been shown in CAD populations with different stresses [20], but data with the triple biomarkers approach in non-ischemic HF are lacking.

### 4.2. Clinical Implications

DSE is feasible in HF and allows a comprehensive risk stratification based upon major determinants of prognosis and functional impairment in HF. The assessment extends beyond the evaluation of RWMA and may include LVCR, CFVR, and HRR with only minimal increase in off-line analysis time for LVCR and on-line imaging time for CFVR. The training and time cost of HRR is negligible. HRR is important prognostic information to predict all-cause death in the long run, and it is also the simplest index to obtain, being independent of imaging, operator, and exercise.

### 4.3. Study Limitations

Not all confounders could be controlled due to observational design. We relied on established criteria for the diagnosis of HF, and they are specific for HFrEF, but less so for HFpEF [1]. CAD was excluded in the majority of patients by invasive or noninvasive coronary angiography and in the remaining patients on the basis of clinical history and symptoms and the absence of inducible RWMA during DSE.

We only assessed CFVR in one coronary artery, since multiple coronary assessment is too complex for routine use. In addition, the coronary microvascular impairment is likely to be diffused and similar in all coronary districts in non-ischemic HF [20].

All-cause death was the primary outcome end-point. In theory, each of the variables under investigation is more closely related to a specific type of death. LVCR is associated to an abnormal myocardial structure and function and therefore may predict HF death. CFVR indicates an abnormal coronary microcirculation and may more likely predict ischemic events. HRR is an index of reduced sympathetic reserve and a predictor of electrical instability, arrhythmias, and sudden death. In practice, it is often difficult to discriminate between cardiovascular and non-cardiovascular causes of death and even more between different causes of cardiovascular death, and therefore, all-cause death is the more robust of all outcome endpoints [21]. However, we complemented this analysis with secondary endpoints cumulating other spontaneous, clinically significant, more frequent end-points. The analysis of composite end-points confirmed the incremental value of each of the three components of the DSE score. As significant endpoints of outcome, we considered not only mortality and HF hospitalization, but also non-fatal myocardial infarction and non-fatal stroke, In fact, HF is a risk factor for stroke even in patients with normal sinus rhythm [22], and myocardial infarction can occur in the follow-up of patients initially considered non-ischemic for a pathogenetic role of coronary microvascular disease in inducing a vulnerability to subsequent myocardial infarction in the presence of angio-graphically insignificant atherosclerosis [23].

The median follow-up period was 20 months, although recruitment in some centers started as early as 2004, since during this long time span some investigators moved or retired and had no more legal access to the updated follow-up information.

## 5. Conclusions

During DSE, patients with non-ischemic HF may frequently show abnormalities of myocardial, coronary microvascular, and cardiac autonomic function. This spectrum of functional defects during stress unmasks a profound prognostic heterogeneity and cannot be predicted on the basis of clinical parameters and resting EF. LVCR, CFVR, and HRR could be included in the evaluation of non-ischemic HF with DSE.

## Figures and Tables

**Table 1:** Study population.

Variable	Overall Population (*n* = 610)	Group 1 with Heart Failure with Preserved Ejection Fraction (HFpEF) (*n* = 270)	Group 2 with Heart Failure with Mid-range Ejection Fraction (HFmrEF) (*n* = 146)	Group 3 with Heart Failure with Reduced Ejection Fraction (HFrEF) (*n* = 194)	*p*
Age (years)	65 ± 11	67 ± 11	64 ± 12	64 ± 12	0.001
Male gender, N (%)	359 (58.9%)	128 (47.4%)	103 (70.5%)	128 (66.0%)	<0.001
BSA (m^2^)	1.87 ± 0.26	1.85 ± 0.26	1.90 ± 0.30	1.87 ± 0.23	0.141
Hypertension, N (%)	388 (63.6%)	204 (75.6%)	100 (68.5%)	84 (43.3%)	<0.001
Diabetes mellitus, N (%)	143 (23.4%)	64 (23.7%)	44 (30.1%)	35 (18.0%)	0.033
Left bundle branch block, N (%)	187 (30.7%)	42 (15.6%)	36 (24.7%)	109 (56.2%)	<0.001
≥Moderate MR, N (%)	106/369 (28.7%)	9/153 (5.9%)	10/64 (15.6%)	87/152 (57.2%)	<0.001
Beta-blockers, N (%)	325 (53.3%)	121 (44.8%)	74 (50.7%)	130 (67.0%)	<0.001
ACE-inhibitors or sartans, N (%)	424 (69.5%)	166 (61.5%)	92 (63.0%)	166 (85.6%)	<0.001
Diuretics, N (%)	249 (40.8%)	47 (17.4%)	44 (30.1%)	158 (81.4%)	<0.001

ACE, angiotensin-converting enzyme; BSA, body surface area; MR, mitral regurgitation.

**Table 2:** Stress echo findings.

	Overall Population (*n* = 372)	Group 1 with HFpEF (*n* = 94)	Group 2 with HFmrEF (*n* = 104)	Group 3 with HFrEF (*n* = 174)	*p*
C step					
Rest EF (%)	46.7 ± 14.0	59.7 ± 5.5	45.1 ± 2.7	29.8 ± 6.5	<0.001
Stress EF (%)	54.7 ± 18.7	71.2 ± 8.6	52.8 ± 8.2	33.2 ± 10.3	<0.001
Rest force (mmHg/mL)	3.0 ± 2.0	4.7 ± 1.7	4.4 ± 1.1	1.1 ± 0.5	<0.001
Stress force (mmHg/mL)	4.0 ± 3.3	6.6 ± 3.1	3.0 ± 1.8	1.2 ± 1.1	<0.001
LV CR	1.24 ± 0.38	1.41 ± 0.39	1.20 ± 0.25	1.02 ± 0.33	<0.001
C-positivity	234 (62.9%)	30 (31.9%)	51 (49.0%)	153 (87.9%)	<0.001
D step					
Rest CFV (cm/s)	29.6 ± 8.4	27.6 ± 8.5	31.0 ± 7.7	31.4 ± 8.3	<0.001
Stress CFV(cm/s)	65.5 ± 22.6	64.3 ± 16.9	68.2 ± 15.9	65.1 ± 22.6	0.108
CFVR	2.24 ± 0.47	2.38 ± 0.39	2.24 ± 0.42	2.06 ± 0.54	<0.001
D positivity	139 (37.4%)	13 (13.8%)	29 (27.9%)	97 (55.7%)	<0.001
E step					
Rest HR (bpm)	70.7 ± 11.3	69.5 ± 10.6	69.3 ± 10.8	73.3 ± 12.2	<0.001
Peak HR (bpm)	88.6 ± 15.0	90.4 ± 16.1	86.9 ± 13.6	87.5 ± 14.1	0.030
HRR	1.26 ± 0.17	1.31 ± 0.18	1.26 ± 0.16	1.20 ± 0.16	<0.001
E-positivity	189 (50.8%)	29 (30.9%)	47 (45.2%)	113 (64.9%)	<0.001
Score 0	200 (32.8%)	147 (54.4%)	41 (28.1%)	12 (6.2%)	<0.001
Score 1	184 (30.2%)	83 (30.7%)	62 (42.5%)	39 (20.1%)	
Score 2	130 (21.3%)	28 (10.4%)	29 (19.9%)	73 (37.6%)	
Score 3	96 (15.7%)	12 (4.4%)	14 (9.6%)	70 (36.1%)	

CFV: coronary flow velocity; CFVR: coronary flow velocity reserve; EF: ejection fraction; HR: heart rate; HRR: heart rate reserve; LVCR: left ventricular contractile reserve.

**Table 3:** Univariate and multivariate predictors of all-cause of death.

	Univariate Analysis		Multivariate Analysis	
	Hazard Ratio (HR; 95% Confidence Interval (CI))	*p*-Value	HR (95% CI)	*p*-Value
Age (yrs)	1.014 (0.984–1.045)	0.357		
Gender (male)	0.776 (0.410–1.469)	0.437		
Hypertension	1.432 (0.762–2.891)	0.264		
Diabetes mellitus	1.403 (0.647–3.042)	0.392		
Abnormal LVEF at rest	1			
Mild reduced LVEF (<50% & ≥40%)	1.661 (0.675–4.088)	0.70		
Reduced LVEF (<40%)	3.179 (1.505–6.716)	0.002		
C positivity (LVCR: <1.1)	2.114 (1117–4.004)	0.022		
D positivity (CFVR: <2.0)	3.082 (1.658–5.728)	<0.001	4.071 (1.643–10.087)	0.002
E positivity (HRR: <1.22)	5.025 (2.321–10.882)	<0.001	1.992 (0.997–3.981)	0.051
Score 0	1			
Score 1	2.216 (0.573–8.575)	0.249		
Score 2	8.799 (2.589–29.904)	<0.001		
Score 3	9.250 (2.628–32.554)	0.001		

**Table 4:** Univariate and multivariate predictors of hard events.

	Univariate Analysis		Multivariate Analysis	
	HR (95% CI)	*p*-Value	HR (95% CI)	*p*-Value
Age (yrs)	1.023 (1.003–1.043)	0.023		
Gender (male)	0.836 (0.563–1.241)	0.375		
Hypertension	0.969 (0.641–1.466)	0.883		
Diabetes mellitus	1.074 (0.685–1.684)	0.756		
Abnormal LVEF at rest	1			
Mild reduced LVEF (<50% & ≥40%)	1.794 (1.081–2.979)	0.024		
Reduced LVEF (<40%)	2.129 (1.341–3.380)	0.001		
C positivity (LVCR: <1.1)	4.051 (2.565–6.400)	<0.001	2.663 (1.630–4.350)	<0.001
D positivity (CFVR: <2.0)	4.382 (2.929–6.554)	<0.001	2.678 (1.709–4.199)	<0.001
E positivity (HRR: <1.22)	3.022 (1.970–4.616)	<0.001	2.397 (1.435–4.004)	0.001
Score 0	1			
Score 1	2.303 (0.955–5.555)	0.063		
Score 2	7.517 (3.344–16.902)	<0.001		
Score 3	13.334 (6.000–29.633)	<0.001		

## Data Availability

The data presented in this study are available on request from the corresponding author. The data are not publicly available due to the privacy law.

## Abbreviations

The following abbreviations are used in this manuscript:

CAD	coronary artery disease
DSE	dipyridamole stress echocardiography
EF	ejection fraction
HF	heart failure
HFmrEF	heart failure with mid-range ejection fraction
HFpEF	heart failure with preserved ejection fraction
HFrEF	heart failure with reduced ejection fraction
LVCR	left ventricular contractile reserve
RWMA	regional wall motion abnormalities
TTE	transthoracic echocardiography
WMSI	wall motion score index
